# Recombination in Positive-Strand RNA Viruses

**DOI:** 10.3389/fmicb.2022.870759

**Published:** 2022-05-18

**Authors:** Haiwei Wang, Xingyang Cui, Xuehui Cai, Tongqing An

**Affiliations:** State Key Laboratory of Veterinary Biotechnology, Harbin Veterinary Research Institute, Chinese Academy of Agricultural Sciences, Harbin, China

**Keywords:** RNA-dependent RNA polymerase, template switching, homologous recombination, copy-back RNA synthesis, defective viral genome

## Abstract

RNA recombination is a major driver of genetic shifts tightly linked to the evolution of RNA viruses. Genomic recombination contributes substantially to the emergence of new viral lineages, expansion in host tropism, adaptations to new environments, and virulence and pathogenesis. Here, we review some of the recent progress that has advanced our understanding of recombination in positive-strand RNA viruses, including recombination triggers and the mechanisms behind them. The study of RNA recombination aids in predicting the probability and outcome of viral recombination events, and in the design of viruses with reduced recombination frequency as candidates for the development of live attenuated vaccines. Surveillance of viral recombination should remain a priority in the detection of emergent viral strains, a goal that can only be accomplished by expanding our understanding of how these events are triggered and regulated.

## Introduction

### Background

RNA viruses cause significant morbidity and mortality to both humans and livestock on a global scale. This extensive group of viruses includes severe acute respiratory syndrome-coronavirus-2 (SARS-CoV-2) (Wang et al., 2020; Zhou et al., 2020), Ebola virus, poliovirus, foot-and-mouth disease virus (FMDV), and porcine reproductive and respiratory syndrome virus (PRRSV), among many others. Due to the low fidelity and lack of proofreading capacity of the virus-encoded RNA-dependent RNA polymerase (RdRp) (Steinhauer et al., 1992), the genetic heterogeneity of RNA virus populations enables efficient selection of progeny virus variants with newly acquired properties (Duffy et al., 2008). Within populations, recombination is a pervasive phenomenon of RNA viruses and an important strategy for accelerating the evolution of RNA virus populations.

Recombination is critical in the biology and evolution of RNA viruses. Accumulated evidence has shown that RNA virus recombination is one of the main approaches to virus rapid adaptation and evolution. However, there is a lack of in-depth understanding of the effects and the methods of studying virus recombination. This review provides a detailed summary of RNA virus recombination.

### Genetic Recombination in RNA Viruses

Recombination in RNA viruses can be defined as an exchange of genetic material between at least two separate viral genomes. Recombination occurs when two or more viruses co-infect a single cell and exchange segments, often giving rise to viable hybrid progeny. To date, recombinant RNA viruses have been found in both positive-strand RNA viruses and negative-strand viruses (Han and Worobey, 2011; Bentley and Evans, 2018). Recombination is more common in positive single-stranded RNA viruses than in negative single-stranded RNA ones (Patiño-Galindo et al., 2021). Occasionally, RNA viruses exploit recombination to acquire host cellular sequences, largely impacting their functionality (Becher and Tautz, 2011; Zou et al., 2019).

Various animal and plant viruses exhibit different forms of recombination, some of which are unique to RNA viruses and different from DNA viruses. Based on the structure of viral RNA molecules and the sites where recombination occurs, RNA virus recombination can be classified into three types: homologous recombination, aberrant homologous recombination, and non-homologous recombination (Lai, 1992a). Homologous recombination occurs between parental RNA molecules that are similar in sequence, with the recombination region being located on the matching, homologous sequences. The resulting recombinant RNA contains the sequence and structure of the parental RNA molecules (Ji et al., 2020; Alatorre-García et al., 2021; Crook et al., 2021). Similarly, aberrant homologous recombination also requires two parental RNA molecules with similar sequences. However, in this case, the recombination event takes place in an unrelated region near the homologous sequence. Aberrant homologous recombination can result in RNA molecules with insertions, deletions, or duplications and, because of this, it often yields defective viral genomes (Mura et al., 2017; Genoyer et al., 2020; Felt et al., 2021). One specific mechanism of aberrant homologous recombination is copy-back RNA synthesis (Sun et al., 2019; Vignuzzi and López, 2019; Janissen et al., 2021), in which the 3′-end of nascent RNA base pairs to an internal position of the same RNA and continues synthesis, deleting the intervening sequences in the process. In contrast with the previous two cases, non-homologous recombination does not depend on sequence homology between the recombined parental RNA molecules, and the selection of recombined sites remains unclear.

Recombination can occur within the same or between different viral subtypes. For example, the hepatitis C virus (HCV) is a positive-sense, single-stranded RNA genome of the *Flaviviridae* family. HCV recombinants could occur at all levels, including between genotypes, between subtypes of the same genotype, and even between strains of the same subtype (González-Candelas et al., 2011), and a similar pattern of recombination has occurred in poliovirus (Xiao et al., 2022). The recombination rates vary markedly for different viral species. For example, positive single-stranded RNA viruses, such as picornaviruses and coronaviruses, show a high frequency of recombination. In contrast, the Zika virus displays a low recombination frequency (Han et al., 2016). Notably, the estimation of RNA virus recombination frequencies should be taken with caution because current RNA recombination assays require virus viability as an endpoint, thus imparting a strong selection and technical bias.

## Consequences of Virus Recombination

Viral RNA recombination facilitates the ontogeny of viral variants with increased virulence and pathogenesis. The emergence of new variants could be the result of selective pressure or a positive selection. The new variant may respond to host immune pressure and develops advantageous mutations or recombination to make it more transmissible in susceptible populations or better able to escape our natural immune responses. Meanwhile, specific variants may be favored by having higher fitness in that context. The variant with higher replicative capacity can be selected and could be less virulent to the host, and the result does not necessarily have to be detrimental to the host. The impact of the new variant on the host may be double-sided. Understanding the propensity for RNA recombination between similar viruses infecting different host species, for example, establishes an important framework in the control of disease spread between endemic regions and could be used to model and predict the risks of future RNA virus infections.

### Expansion of Host Species or Cell Tropism

**FIGURE 1:**
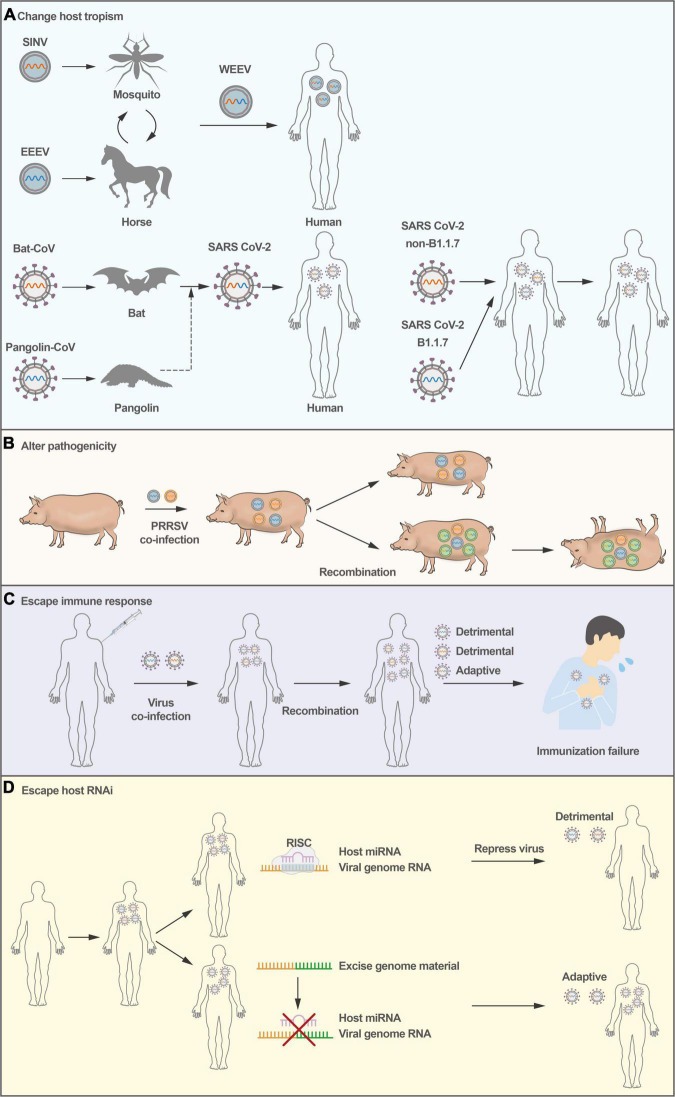
Consequences of virus recombination. **(A)** Virus recombination causes host species expansion; WEEV and SARS-CoV-2 were transferred from animals to humans *via* recombination. **(B)** PRRSV recombination causes pathogenicity alterations in pigs. **(C)** Virus recombination makes the virus escape immunity, and the replication of the virus in the host eventually results in immunological failure. **(D)** RNA viruses could excise genomic material through recombination as an intrinsic defense against antiviral RNA interference.

Accumulating evidence supports the hypothesis that recombination could result in an expansion of cellular tropism and even host tropism. For example, studies have suggested that Western equine encephalitis virus (WEEV), a zoonotic virus that causes encephalitis in humans and severe diseases in horses, seems to be the result of the recombination between an Eastern equine encephalitis virus (EEEV)-like virus and a Sindbis-like virus (Figure 1A), exhibiting the encephalitogenic properties of EEEV and the antigenicity of Sindbis virus (Hahn et al., 1988; Forrester et al., 2012).

More recently, the SARS-CoV-2 pandemic has resulted in high morbidity and mortality in the human population. Despite extensive research, the origin of SARS-CoV-2 remains unclear. The receptor-binding domain (RBD) in its spike protein is almost identical in sequence to that of Pangolin-CoV (Zhang et al., 2020), suggesting that SARS-CoV-2 could have originated from recombination between coronaviruses with different host tropism (Lam et al., 2020; Figure 1A). Notably, genomic recombination analyses have shown that the circulating strains of SARS-CoV-2 from late 2020 and early 2021 in the United Kingdom are inter-lineage recombinants (Jackson et al., 2021). Subsequently, the SARS-CoV-2 epidemic has been under several distinct waves. The first was associated with a mix of SARS-CoV-2 lineages, while the second and third waves were driven by the Beta (B.1.351) and Delta (B.1.617.2) variants (Tegally et al., 2021a,b,c). In November 2021, a new SARS-CoV-2 variant associated with a rapid resurgence of infections was detected, and it was designated a variant of concern (Omicron, B.1.1.529) (Viana et al., 2022). The Omicron has three major lineages BA.1, BA.2, and BA.3. Given a large number of mutations between BA.1, BA.2, and BA.3 and other known SARS-CoV-2 lineages, it is thought that this may be associated with recombination. However, weak statistical and phylogenetic evidence could be used to determine the recombination of BA.1, BA.2, or BA.3 (Viana et al., 2022). Moreover, recombinant viruses are products that could be under a series of multiple partially overlapping recombination events. The existing evidence can not fully prove the complex recombination patterns.

Recombination in beta coronaviruses, including natural populations of MERS-CoV (Dudas and Rambaut, 2016; Kim et al., 2016; Schroeder et al., 2021), SARS, and SARS-like coronaviruses (Hon et al., 2008; Boni et al., 2020), occurs with increased frequency, and the recombinant viruses tend to be highly adaptable. RNA recombination allows viruses to drive the generation of new viral variants to be able to adapt to hosts in nature. Such recombination events may result in the emergence of a viable and highly virulent virus with altered tropism restrictions. In addition, recombination events could lead to exchangeable neutralization epitopes, facilitating the immune escape of highly contagious recombinant viruses in previously protected populations (Walsh et al., 2009).

### Emerging Virulent Variants

Recombinant viruses can preserve or increase the pathogenicity of the parental strains (Shi et al., 2013; Mandary and Poh, 2018). For instance, etiological investigations for an outbreak of paralytic poliomyelitis that occurred in the Dominican Republic and Haiti between 2000 and 2001 revealed that the responsible viruses were recombinants between the poliovirus Sabin 1 strain and species C enteroviruses (Kew et al., 2002). In addition, a poliomyelitis outbreak was caused by type 2 vaccine-derived poliovirus recombinants in the Central African Republic in 2019 (Joffret et al., 2021). Finally, hand, foot, and mouth disease (HFMD) is usually a mild childhood disease caused mainly by coxsackievirus A16 and enterovirus A71 (EV-A71). In 2018, intra- and inter-typic recombination events amongst co-circulating EV-A71 strains contributed to the outbreak and epidemic of HFMD in the Western Pacific countries (Ang et al., 2021).

Virulent variants of animal viruses can be generated by recombination. Recombination occurs between wild-type PRRSV strains and between the modified live vaccine (MLV) strain and wild-type PRRSV (Zhou et al., 2018b; Wang et al., 2019). In 2009 and 2010, there was a marked increase in PRRSV infections in China, and the viruses for this outbreak were found to originate from a single recombination event (Shi et al., 2013). In 2013 and 2014, a recombinant NADC30-like PRRSV strain emerged, the product of recombination between a North America PRRSV NADC30 strain and a local strain in China, which caused 76.7% deaths in a pig farm and showed higher pathogenicity than its parental NADC30 PRRSV strain (Zhao et al., 2015; Figure 1B). More importantly, recombination between two MLV PRRSV strains produced pathogenic PRRSV, leading to disease outbreaks in many pig farms in France and Denmark (Eclercy et al., 2019; Kvisgaard et al., 2020). In addition, virulent recombinants have also been observed in cases of FMDV (Abd El Rahman et al., 2020), enterovirus EV-B83 (Xiao et al., 2020), and rodent coronavirus (Li et al., 2021). In addition, RNA virus recombination can significantly accelerate adaptation and virus spread, dramatically enhancing virulence (Xiao et al., 2016). Novel emerging virulent recombinants make these new strains more complex than their parental strains, posing many challenges in the prevention of epidemic diseases.

### Host Selection and Virus Fitness

Recombination can create considerable changes in the viral genome, allowing for antigenic shifts, host jumps, and fitness modifications (Simon-Loriere and Holmes, 2011). For viruses with large genomes, the fitness of novel recombinant viruses may be particularly susceptible to the effects of recombination (Pérez-Losada et al., 2015). For example, the spike glycoprotein of coronaviruses has previously been identified as a recombination hotspot (Graham and Baric, 2010). Recombinants with new spikes may broaden or alter receptor usage, enabling host switches or expanding their host range (Goldstein et al., 2021). Chikungunya virus (CHIKV), an important human pathogen that has caused a long history of epidemics in Africa and Asia, has rapidly spread in Europe and the Americas (Lanciotti and Valadere, 2014). RNA copy-choice recombination of CHIKV is responsible for genome diversification. Moreover, RNA recombination can generate new viral variants that are positively selected in mosquito vectors, working as an efficient strategy to allow CHIKV to overcome “tight” genetic bottlenecks or even providing an advantage for viral replication (Filomatori et al., 2019). For poliovirus, a virus with a significantly smaller genome than coronavirus, recombination accelerates adaptation in the short time frame of acute poliovirus infection and enriches beneficial mutations while purging deleterious mutations (Xiao et al., 2016). Two high-frequency inter-lineage recombination hotspots have been located in the RdRp and GP2–GP3 regions of PRRSV-2 (Zhou et al., 2018a). The RdRp is involved in PRRSV replication, while GP2–GP4 are the major determinants of virus entry into host cells (Das et al., 2010; Liu et al., 2016; Zhao et al., 2018). The recombination hotspots of PRRSV-2 might be associated with an increase in replication capacity and cellular tropism, which presumably contributes to virus survival and interhost spread.

### Failure of Vaccines Protection

Recombination can generally generate virus variants to be able to escape host immune responses. For example, poliovirus recombinants have been isolated from vaccine-associated poliomyelitis patients. Certain recombinants, particularly between type 3 and type 2 poliovirus vaccine strains, are frequently isolated (Minor et al., 1986). Recombination contributes to fitness and virulence in circulating vaccine-derived polioviruses (cVDPVs), just as it does for wild-type viruses. However, some recombinant cVDPVs do not escape host immune responses more than wild-type poliovirus. In addition, highly pathogenic porcine epidemic diarrhea virus (PEDV) strains emerged in China in 2012, circumventing existing vaccines, and RNA recombination events between wild-type and live-attenuated PEDV have seeded new outbreaks (Sun et al., 2012). The recombinant virus can escape from host adaptive immune responses, leading to failure in vaccine protection (Figure 1C). Therefore, RNA virus recombination could pose a potentially serious problem in the administration of MLV vaccines. For poliovirus, adaptive alleles can rapidly accumulate with effective recombination to alleviate the deleterious mutational load. Changing the adaptability may alter virus infection, which presents several possibilities for therapeutic strategies and rational vaccine design (Xiao et al., 2017).

### Generation of Recombination Deficient Viruses

Accumulating evidence has shown that RNA recombination occurs at specific RNA secondary structures or homologous sequences between the two parental viruses (Rowe et al., 1997; Chuang and Chen, 2009; Runckel et al., 2013). Therefore, recombination rates could be reduced by altering stem-loop sequences or polymerase fidelity, generating recombination-deficient viruses with the potential for vaccine candidates. Some non-recombinogenic vaccines are developing, such as poliovirus type 2 vaccines. Yeh et al. (2020) introduced modifications within the 5′ untranslated region (UTR) of the Sabin 2 genome to stabilize attenuation determinants, 2C coding region to prevent recombination, and 3D polymerase to limit viral adaptability. Nonetheless, recombination deficient viruses may exhibit lower fitness. Thus, RNA recombination could produce selective disadvantages in progeny viruses, and care must be taken when interpreting such results.

### Evasion of Host RNA Interference Defense Mechanism

In addition to the host interferon system combating viruses early upon infection, vertebrate organisms employ RNA interference (RNAi) as an antiviral defense mechanism. In this regard, positive-stranded RNA viruses can undergo strand-switching to rapidly excise genomic material through recombination as an intrinsic defense against antiviral RNAi (Aguado et al., 2018), rendering the virus capable of evading host RNAi-mediated defenses (Figure 1D). The rapid recombination capacity of positive-stranded RNA viruses provides them a unique feature that would significantly enable diversity in the context of evading RNAi, in addition to other innate cellular responses. There is some literature suggesting that RNAi is an antiviral mechanism for invertebrate hosts (Lecellier et al., 2005; Triboulet et al., 2007; Castanotto and Rossi, 2009), and plays a crucial role in the antiviral defense of invertebrates. However, the contribution of RNAi to mammalian antiviral innate immune defense has been underestimated and disputed, and more research may be needed in the future.

## The Mechanisms of RNA Virus Recombination

### Replicative and Non-replicative Mechanisms of RNA Virus Recombination

**FIGURE 2:**
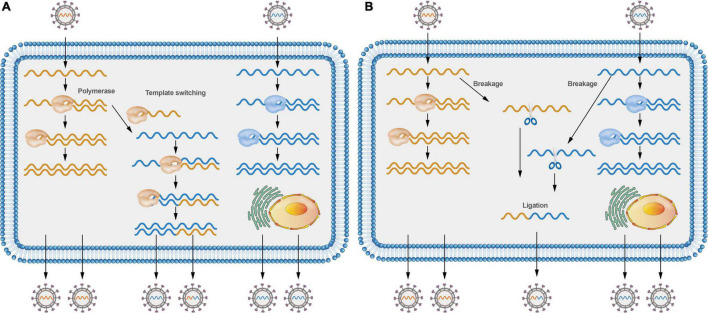
Models of positive-sense RNA viruses’ recombination. **(A)** Different viruses co-infecting a cell can switch viral templates, and RdRP can switch from donor template to receptor template to generate recombinant molecules. **(B)** Different viruses co-infecting a cell can lead to virus RNA breakage; RNA molecules can be cut at specific sites and then joined to form recombinant molecules.

Various molecular mechanisms have been proposed to describe the recombination events in RNA viruses. To date, both the replicative copy-choice model and the non-replicative breakage-rejoining model have gained widespread acceptance. Most RNA viruses recombine through a copy-choice mechanism, which involves the formation of recombinant RNA molecules when the RdRp switches from one template to another during genome replication (Figure 2A; Lai, 1992b). However, whether the RdRp switches from one template to another is still a debatable issue. A new RdRp may engage the nascent RNA products after they align on a new template. Non-replicative recombination, a different process in which RNA molecules are cut at specific sites and then joined to form recombinant molecules (Figure 2B), has also been demonstrated in multiple viruses (Becher and Tautz, 2011; Holmblat et al., 2014; Imai et al., 2019), such as recombination between defective poliovirus and coxsackievirus genomes. Non-replicative recombination events may be a transient, intermediate step in the generation and selection of the fittest recombinants.

### Polymerase Fidelity Influences Viral Recombination

**FIGURE 3:**
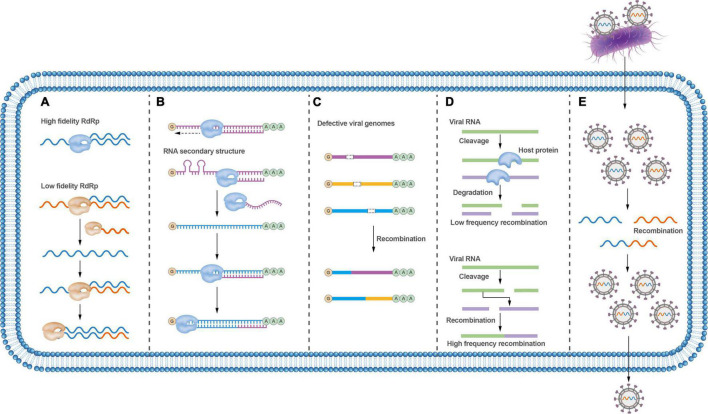
Factors that affect RNA virus recombination. **(A)** RdRp fidelity affects the recombination rate; low-fidelity RdRp variants are more prone to recombination. **(B)** Secondary RNA structures between distinct viruses may block strand-switching and lead to virus recombination. **(C)** Defective viruses can undergo recombination to exchange gene fragments to require the whole viral genome; viruses can recombine with unrelated viral genes or cellular genes. **(D)** Host proteins may influence virus recombination. **(E)** Bacteria can carry viruses during co-infection, thus facilitating virus recombination.

The viral RdRp plays a central role in mediating template-switching recombination. The fidelity of viral RdRp has shown to be a fundamental determinant of recombination frequency in both cell-based and biochemical assays (Fitzsimmons et al., 2018). Viral RdRp with low fidelity displays higher recombination frequencies, while viral RdRp with higher fidelity shows lower recombination frequencies (Poirier et al., 2015; Figure 3A). Interestingly, it has been shown that a ribavirin-resistant mutant (with a high-fidelity polymerase) rendered a virus resistant to ribavirin-induced error catastrophe only when recombination occurred at wild-type levels; and, accordingly, this variant became susceptible to ribavirin-induced error catastrophe when recombination was impaired (Kempf et al., 2019). These results support that recombination counteracts the negative consequences of replicative misincorporation, likely by purging genomes harboring deleterious mutations through recombination events. This process is consistent with a previously proposed model for recombination in enteroviruses (Lowry et al., 2014), a biphasic replicative process involving the generation of greater-than genome length “imprecise” intermediates followed by deletion of additional RNA sequences to maximize viral fitness. Whether RdRp errors trigger recombination is disputable.

### Nucleotide Sequences Affect Viral Recombination

Nucleotide homology, nucleotide composition, and secondary structures of viral genomic sequences could all affect the frequency of recombination (Marquez-Jurado et al., 2015). Recombination analysis of PRRSV revealed that most recombinations take place in regions with high genetic identity, and intra-lineage recombination is more frequent than that within inter-lineage (Das et al., 2010). For brome mosaic virus (BMV), the recombinant breakpoints cluster within or close to AU-rich regions (Nagy and Bujarski, 1997), and it is thought that the RdRp likely stops at an AU-rich location in the template chain and then jumps to another template chain during viral replication (Simon-Loriere and Holmes, 2011). Nucleotide composition has also been found to play an important role in determining the sites of homologous recombination of poliovirus (Runckel et al., 2013).

RNA secondary structures were confirmed to convey critical functional information during the recombination process (Figure 3B). It has been demonstrated that the recombination hot spots are located around hairpin structures in the genome of the turnip crinkle virus (TCV). Such RNA secondary structures are thought to inhibit the movement of RdRp and promote its switch between templates (Carpenter et al., 1995; Nagy et al., 1999). Similarly, gene synthesis and deep sequencing analysis of the breakpoints throughout the poliovirus genome have revealed that most breakpoints are associated with RNA secondary structures, including hairpins and stem-loop structures (Runckel et al., 2013). RNA secondary structures also seem to promote recombination in HIV and increase the viability and replication ability of recombinant HIV progeny (Simon-Loriere et al., 2010). Nonetheless, a recent study argues that recombination is not influenced by the RNA donor or acceptor sequence or its structure (Alnaji et al., 2020), which seems to contradict previous studies that recombinant virus genomes are related to RNA structure and sequence identity. Importantly, more recent research indicates that viruses undergo frequent and continuous recombination events over a prolonged period until the fittest and wild-type length of the recombinant genome is dominant (Bentley et al., 2021). These conflicting results may reflect variation in the recombination processes involved between different viruses. In the meantime, it also supports that recombination is a promiscuous event that is not significantly influenced by any single factor.

### The Effects of Host or Bacteria Factors on Viral Recombination

Virus recombination can be affected by hosts. Several plant viruses, including TCV, tomato bushy stunt virus, cucumber necrosis virus, and potato virus X, have been shown to undergo non-homologous RNA recombination between a defective viral RNA and a helper wild-type virus RNA (Sztuba-Solińska et al., 2011; Figure 3C). As such, the host and other extrinsic factors can play important roles in this type of recombination. Host factors, combined with environmental factors that affect the Met22/Xrn1 pathway, seem to also play a role in viral RNA recombination (Jaag and Nagy, 2010). Host cellular RNA helicase RH20 and proteasomal Rpn11 protein can affect RNA recombination and viral replication (Prasanth et al., 2015; Wu and Nagy, 2020; Molho et al., 2021; Figure 3D).

Nidoviruses, including SARS-CoV-2, exhibit discontinuous transcription and produce a set of sub-genomic RNAs that contribute to high recombination rates (Yang et al., 2021). These viruses can incorporate either unrelated viral genes or cellular genes into the viral genome, possibly by a non-homologous recombination mechanism. Occasionally, cellular genes have been incorporated into viral genomes, such as the ubiquitin gene, which is regularly incorporated into the genome of bovine viral diarrhea virus, contributing to the cytopathogenic function displayed by the virus (Meyers et al., 1991).

In addition, bacterial infection may also affect viral recombination. The ability of enteric RNA viruses to undergo homologous recombination is variable and depends, at least in part, on bacterial infections that facilitate co-infection of different viral genotypes within the same cell (Erickson et al., 2018; Figure 3E).

## Development of Assays to Quantify Virus Recombination Rates

### Plaque-Purification and DNA Sequencing

Researchers often used a method of plaque purification to isolate recombinant viruses. For example, the investigation of intra- and inter-typic recombinant crosses of poliovirus was possible by this approach, as they were able to produce plaques *in vitro* (Kirkegaard and Baltimore, 1986). Such recombinants with a growth or plaque formation advantage can often be easily identified and differentiated from the two parental viruses.

For feline calicivirus, recombination rates have been detected in co-infected cells using quantitative reverse transcription-polymerase chain reaction amplification (RT-qPCR) (Symes et al., 2015). This method utilizes virus-specific primers upstream and downstream of the recombination hotspot that hybridized with only one of the strains during the co-infection, and the site of recombination is then confirmed by DNA sequencing. A similar approach has been used to detect recombination in poliovirus (Jarvis and Kirkegaard, 1992). The direct and quantitative detection of recombinant RNA molecules by PCR allows the study of recombination in RNA viruses without the requirement of a viable progeny, an endpoint that can bias the interpretation of predictive conclusions.

The above approach is a more classic recombination detection method, and while the results are more accurate, the process is time-consuming with a huge workload.

### Cell-Based Recombination Assay

A cell-based recombination assay (termed CRE-REP) established by the Evans laboratory has drastically advanced studies of enterovirus recombination (Lowry et al., 2014). This approach is based on the co-transfection of two *in vitro* transcribed RNAs that alone cannot produce viable viruses. In this system, the “donor” RNA is a replication-competent, sub-genomic RNA encoding a luciferase reporter gene in place of the viral capsid sequences. In turn, the “acceptor” RNA is a replication-incompetent, genomic RNA that has a defective *cis*-acting replication element, and only after successful recombination between these two parental genomes, viable progeny recombinant viruses can be produced and quantified.

Since its emergence, this technique is currently extensively utilized for virus recombination studies in the poliovirus (Kempf et al., 2016; Kim et al., 2019; Woodman et al., 2019). Researchers have further adapted the CRE-REP assay, making it suitable for other viruses, such as the Seneca Valley virus (SVV) (Li et al., 2019). For instance, a lethal catalytic mutation can be introduced into the RdRp active site (Kempf et al., 2016; Li et al., 2019), the active site of the RdRp can be removed from the “acceptor” R NA (Woodman et al., 2019), or a lethal capsid deletion of the “donor” RNA (Kempf et al., 2016; Li et al., 2019) can be used to make both parental genomes non-viable. In addition, the use of fluorescent proteins as reporter genes (Kim et al., 2019) has enabled researchers to visualize and quantify viruses that are unable to form plaque efficiently by monitoring recombination events through imaging or cell sorting. CRE-REP assay is a particularly sophisticated method for detecting virus recombination at the cellular level. However, only some enteroviruses belonging to the family *Picornaviridae* have used this kind of assay to identify the recombination rate. Whether this method can be applied to other virus families has not been investigated.

### *In vitro* Biochemically Defined Recombination Assay

A recent study of poliovirus recombination utilizes two different biochemical assays to distinguish copy-choice recombination from forced copy-choice recombination (Kim et al., 2019). In both assays, the purified RdRp engages a primed template, elongates that primer, disengages from the first template, engages a second (acceptor) template, and continues RNA synthesis. One assay uses oligo (dT) or oligo (rU) to prime poly (rU) RNA synthesis on oligo (rA) or poly (rA) RNA templates to mimic copy-choice recombination, which was first used to show template-switching of the RdRp during elongation (Arnold and Cameron, 1999). The RdRp-nascent RNA complex then moves to a new template from internal positions or, as a consequence of reaching the end of the template, creates greater-than-template-length products. The second assay uses a heteropolymeric, symmetrical, primed-template substrate (sym/sub) and is based on template-switching from the end of the template, thus mimicking forced copy-choice recombination (Woodman et al., 2016). This method can analyze recombination qualitatively and quantitatively in a precise and intuitive manner. However, performing these biochemical tests can be technically challenging.

### Third-Generation Sequencing Approach Combined Sequence Analysis

Recent advances in genome sequencing by third-generation sequencing technologies (TGS), including PacBio single-molecule real-time sequencing and nanopore sequencing technology, can generate reads of >10 kb in length (Lu et al., 2016; Magi et al., 2017). The long reads enable to distinguish the recombinant viruses from the mixed parental viruses. A direct RNA sequencing approach based on nanopore sequencing technology has been used to characterize viral RNAs produced in cells infected with the HCoV-229E coronavirus, and several unexpected recombination sites were observed in the ORF1a 5′- and 3′-terminal regions (Viehweger et al., 2019). Although TGS showed a high error rate compared to next-generation sequencing (NGS) or Sanger methods, most of the errors in the assay were nucleotide-substitutions that did not affect recombination analysis. In addition, Tiled-ClickSeq has been developed to sequence the complete genome coverage, including the 5′UTR, at high depth and specificity to the virus on both Illumina and Nanopore platforms (Jaworski et al., 2021). Besides, a direct RNA sequencing approach was performed with samples from total RNA obtained from virus-infected cells where there is no bias from reverse transcription and amplification, allowing the quantification and analysis of the numbers of recombinant or parental molecules (Viehweger et al., 2019).

With the advancement and maturation of sequencing technology, an increasing number of experiments are opting for third-generation sequencing for the research. Although third-generation sequencing is certainly speedy and convenient for recombinant virus analysis, its high cost and greater error rate have led to some laboratories using Sanger sequencing instead.

### Bioinformatics Assay

Bioinformatics analysis of genomic sequence data contributes to an in-depth understanding of the evolutionary process, including the impact of genomic structure (Reich et al., 2001), phenotypic variation (Zhang et al., 2002), and its relationship with genetic disease research (Daly et al., 2001). RNA virus recombination is one of the most important aspects of evolutionary biology, and bioinformatic methods can aid in adequately detecting its occurrence and understanding how it influences phylogenetic relationships (Posada et al., 2002). Phylogenetic tree analysis is generally a bioinformatics method to evaluate virus recombination (Posada et al., 2002). In addition, a phylogenetic compatibility matrix (PCM) for different regions of the enterovirus genome and correlations with sequence assignments to analyze recombination among complete genome sequences can be used (Simmonds and Welch, 2006). Determination of the variation in viral recombination and whether they are related to other biological characteristics is essential for future research. With the development of sequencing technology, the application of high-throughput sequencing technology combined with biological analysis is also a common method to analyze recombination. In the recombination analysis of SARS-CoV-2, Illumina and Nanopore reads and Python scripts were used to analyze the data and map RNA recombination events (Jaworski et al., 2021). Besides, sequences obtained from NGS and bioinformatics tools were used to analyze the recombination events within enterovirus C serotypes (Amona et al., 2020).

## Summary and Outlook

Most RNA viruses can recombine either by homologous or non-homologous recombination, both of which play an important role in the biology and evolution of RNA viruses. Recombination is a major driving force for viral diversity and has significant biological consequences on viral biology. Recombination generates novel viral strains, which expands virus genetic diversity, increases virulence and pathogenesis, and promotes the emergence of new circulating viruses. In addition, recombination can facilitate the evolution of antiviral resistance, host immunity escape, and associate with alterations or expansions of viral host tropism. Thus, it is important to emphasize that viral recombination detection and surveillance need to be prioritized for viral disease control and prevention. Understanding the mechanisms of virus recombination and the factors that affect recombination can ultimately help us design live attenuated vaccines unable to recombine with circulating strains.

Simultaneously, recombination can be used as a beneficial tool for research. Reporter viruses are useful tools for screening susceptibility and resistance to antiviral compounds. However, the stability of reporter viruses has been challenged by viral RNA recombination, leading to the deletion of the reporter gene during viral replication. Lethal mutations upon recombination have been engineered to create a stable Zika virus harboring a NanoLuc-reporter gene (Baker et al., 2020). The developed recombination-dependent lethal approach could produce stable reporter viruses used for rapid diagnosis, vaccine evaluation, and antiviral discovery.

## Author Contributions

TA and XHC contributed to conception of the study. HW and XYC wrote the draft. TA revised the draft. All authors read and approved the final manuscript.

## Conflict of Interest

The authors declare that the research was conducted in the absence of any commercial or financial relationships that could be construed as a potential conflict of interest.

## Publisher’s Note

All claims expressed in this article are solely those of the authors and do not necessarily represent those of their affiliated organizations, or those of the publisher, the editors and the reviewers. Any product that may be evaluated in this article, or claim that may be made by its manufacturer, is not guaranteed or endorsed by the publisher.
